# *In Vitro* Fertilization and Embryo Culture Strongly Impact the Placental Transcriptome in the Mouse Model

**DOI:** 10.1371/journal.pone.0009218

**Published:** 2010-02-15

**Authors:** Patricia Fauque, Françoise Mondon, Franck Letourneur, Marie-Anne Ripoche, Laurent Journot, Sandrine Barbaux, Luisa Dandolo, Catherine Patrat, Jean-Philippe Wolf, Pierre Jouannet, Hélène Jammes, Daniel Vaiman

**Affiliations:** 1 Service d'Histologie-Embryologie, Biologie de la Reproduction, Hôpital Cochin, Paris, France; 2 Université Paris Descartes, Paris, France; 3 Département Génétique et Développement, Equipe 21, Institut Cochin, INSERM U567, CNRS (UMR 8104), Paris, France; 4 Plateforme de Génomique, Institut Cochin, Paris, France; 5 Institut de Génomique Fonctionnelle, Montpellier, France; 6 Biologie du Développement et Reproduction, UMR1198 INRA-ENVA, Jouy en Josas, France; Ottawa Hospital Research Institute and University of Ottawa, Canada

## Abstract

**Background:**

Assisted Reproductive Technologies (ART) are increasingly used in humans; however, their impact is now questioned. At blastocyst stage, the trophectoderm is directly in contact with an artificial medium environment, which can impact placental development. This study was designed to carry out an in-depth analysis of the placental transcriptome after ART in mice.

**Methodology/Principal Findings:**

Blastocysts were transferred either (1) after *in vivo* fertilization and development (control group) or (2) after *in vitro* fertilization and embryo culture. Placentas were then analyzed at E10.5. Six percent of transcripts were altered at the two-fold threshold in placentas of manipulated embryos, 2/3 of transcripts being down-regulated. Strikingly, the X-chromosome harbors 11% of altered genes, 2/3 being induced. Imprinted genes were modified similarly to the X. Promoter composition analysis indicates that FOXA transcription factors may be involved in the transcriptional deregulations.

**Conclusions:**

For the first time, our study shows that *in vitro* fertilization associated with embryo culture strongly modify the placental expression profile, long after embryo manipulations, meaning that the stress of artificial environment is memorized after implantation. Expression of X and imprinted genes is also greatly modulated probably to adapt to adverse conditions. Our results highlight the importance of studying human placentas from ART.

## Introduction

It is estimated that 2.2% of children born each year in the United States were conceived with the help of assisted reproductive techniques (ART) including *in vitro* fertilization (IVF) and intra-cytoplasmic sperm injection (ICSI) [1]. Although these techniques are thought to be safe, new evidence suggests an increased risk of morbid congenital problems associated with their use [2], [3], such as cerebral palsy and delayed development [4], low birth weight [5], [6], and increased major birth defects [3], [5]–[9]. Furthermore, in recent years even if contradictory findings exist [9]–[12], several reports suggested that IVF increases the risk of epigenetic disorders [13], [14] including Beckwith–Wiedemann [15]–[19], Angelman [18], [20], [21] and Silver-Russell syndromes [22]–[24].

While infertility, *per se*, could be at least partly responsible for these diseases [25], the extent of the anomalies observed indicates that infertility alone does not explain all the alterations, part of them being certainly due to embryo culture and more generally to the reproductive technology applied to the gametes and the embryo. Indeed, studies in animal models have demonstrated that embryo culture [26], [27] and *in vitro* fertilization [28], [29] of mouse or bovine preimplantation embryos alter their gene expression patterns when compared with *in vivo* produced embryos. It is important to investigate whether these changes are only temporary or whether they may subsequently induce deleterious consequences. In mice, post-natal development and behavioral parameters (memory deficiencies at adult stage, altered behaviors and growth anomalies in standard tests) have been shown to be affected by embryo culture during the preimplantation period [30], [31]. These abnormal phenotypes could be the consequences of early (around implantation) gene profile modifications with persistent effects.

**Figure 1:**
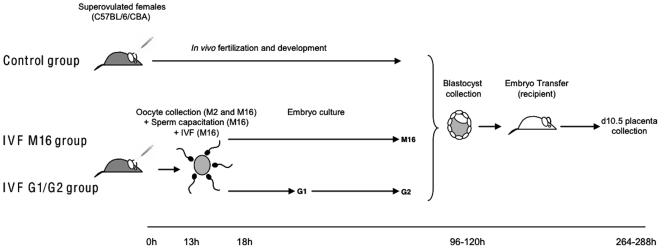
Experimental design. *In vivo* fertilization and preimplantation development (Control group) and *in vitro* fertilization and preimplantation embryo development (IVF groups). Oocyte collection was performed in M2 and then in M16. Sperm capacitation and the fertilization step were conducted in M16. Embryo culture was performed in two different culture media: M16 and G1/G2. The timeline in hours (h) is relative to the hCG injection (0 h). For all groups, the blastocysts were transferred in pseudopregnant d3.5 F1 females.

The placenta plays a crucial developmental function as the interface between the mother and the developing fetus. As such, it has been proposed to play the role of a filter against deleterious environmental influences, such as exposure to xenobiotics [32], toxins [33], [34], infectious agents and parasites [35]. In the present study, we analyzed the transcriptomic effects of *in vitro* embryo manipulations on the expression profile of placental genes (analyzed at 10.5 days post-fertilization, as depicted in Figure 1). We show that early manipulations are not completely buffered at post-implantation stage. In fact, roughly 6% of the transcripts are altered at the threshold of two-fold in placentas of manipulated embryos compared with placentas of embryos produced *in vivo*. In addition, we show by bioinformatics promoter analysis that specific transcription factors could be involved in these deregulations. Finally, we show that many imprinted genes known to be crucial for placental development [36], [37], are more deregulated than the rest of the transcriptome, and overall in the up-regulation direction contrarily to the rest of the genome. Many of these deregulated imprinted genes belong to the previously identified imprinted gene network [38], and some were previously found modulated by ART [14]; Fauque et al., submitted). Interestingly, we observe that like imprinted genes, genes from the X chromosome behave similarly to imprinted genes following IVF manipulations. We discuss the potential impact of these targeted modifications.

## Results

### IVF and Embryo Culture Trigger Substantial Transcriptional Modifications on Placental Gene Expression Profiles

**Figure 2:**
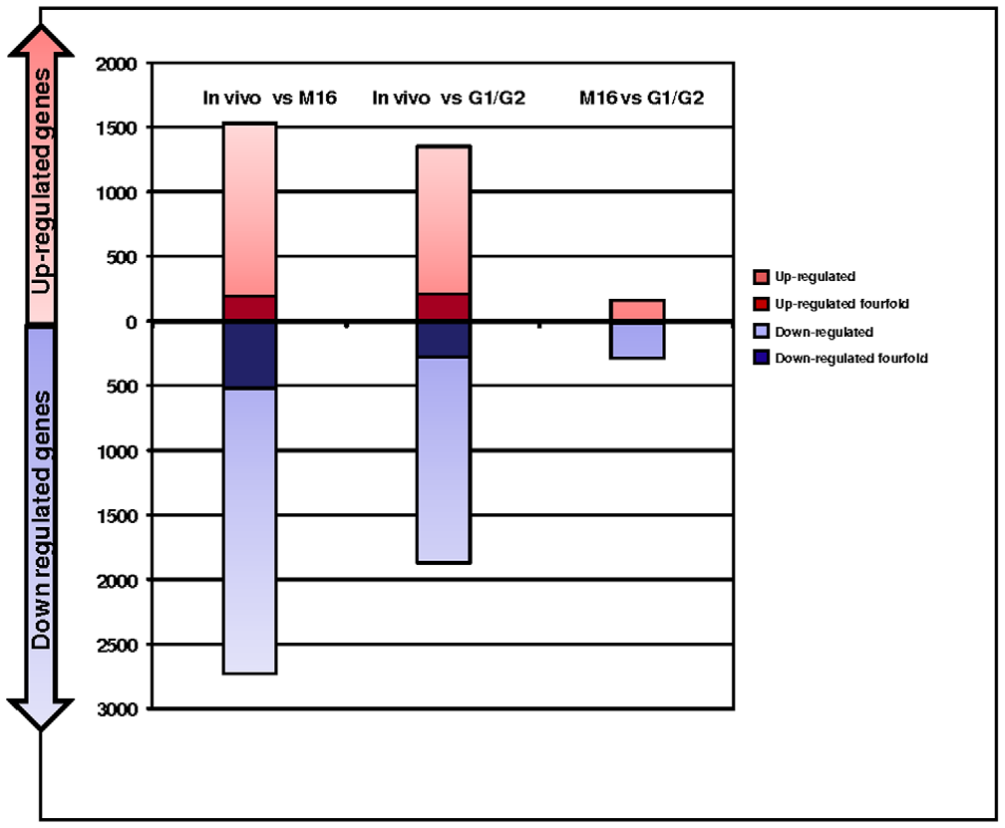
Number of transcripts differentially regulated according to the conditions of fertilization and early embryo development. The number of transcripts either up- (red) or down-regulated (blue) represents the number of transcripts changed two-fold (pale color) or four-fold (dark color) after comparison of the IVF groups (IVF M16 and IVF G1/G2 groups) versus the control group.

**Figure 3:**
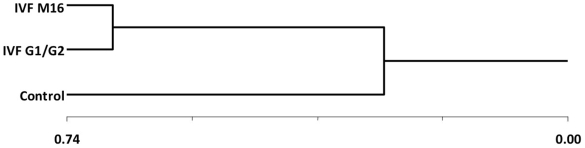
Hierarchical clustering analysis according to the different procedures (IVF M16 and G1/G2 groups and control group).

The expression of placental genes was compared between placentas from the different groups (Figure 1). The Affymetrix 430 2.0 microarray that was used encompasses a complete set of mouse transcripts (45,101 transcripts). We calculated a ratio of modification for each gene, by pairwise comparisons between the three conditions (*in vivo*, *in vitro* M16, and *in vitro* G1/G2), in order to count how many genes were modified at the two-fold or four-fold thresholds (either up- or down-regulated), as shown in Table 1 and Figure 2. At the two-fold threshold, *in vitro* fertilization and embryo culture in M16 resulted in the overall deregulation of 3,553 transcripts compared to *in vivo* produced embryos, while G1/G2 culture induced the expressional alterations of 2,721 transcripts. At the four-fold threshold, the number of modified transcripts was reduced more than five times, with 703 and 494 misregulated transcripts after culture in M16 and G1/G2, respectively. Overall, the count of dysregulated transcripts after *in vitro* fertilization and embryo culture versus *in vivo* conditions was much higher than when the two *in vitro* conditions of embryo culture were compared (Table 1). The same idea is illustrated when all the transcripts were taken into consideration by a hierarchical clustering analysis of the expression profiles. This representation shows a striking separation into two major branches opposing the *in vivo* sample to the two *in vitro* samples (correlation coefficient between IVF M16 and IVF G1/G2  = 0.67, Figure 3). IVF M16 and IVF G1/G2 samples were further divided into two little branches (correlation coefficient IVF M16 versus control  = 0.14 and IVF G1/G2 versus control  = 0.27).

**Table 1:** Number of modified placental transcripts at the two-fold and four-fold thresholds.

	IV vs M16	IV vs G1/G2	M16 vs G1/G2
> two-fold up-regulated	1340 (3.0%)	1135 (2.5%)	157 (0.3%)
> two-fold down-regulated	2213 (4.9%)	1586 (3.5%)	276 (0.6%)
> four-fold up-regulated	191 (0.4%)	217 (0.4%)	6 (0.0%)
> four-fold down-regulated	512 (1.1%)	277 (0.6%)	11 (0.0%)

In addition, it was interesting to note that most genes modified by *in vitro* culture were down-regulated. This down-regulation was stronger with M16 than with G1/G2 (Figure 2).

### Modified Genes Are Involved in Cell Growth, Protein Modifications, Immune Regulation, Complement and Coagulation, and Angiogenesis

After observing very few differences between the two media used for embryo culture, we performed functional clustering analyses by comparing control samples and samples from culture carried out in the G1/G2 sequential medium. The major reason for this choice was the fact that G1/G2 is one of the most classical medium used in human ART. The results are slightly milder than when M16 was used; however, they most generally occur in the same direction. For this analysis, we focused exclusively on genes (one gene being sometimes represented in the array by more than one transcript).

**Figure 4:**
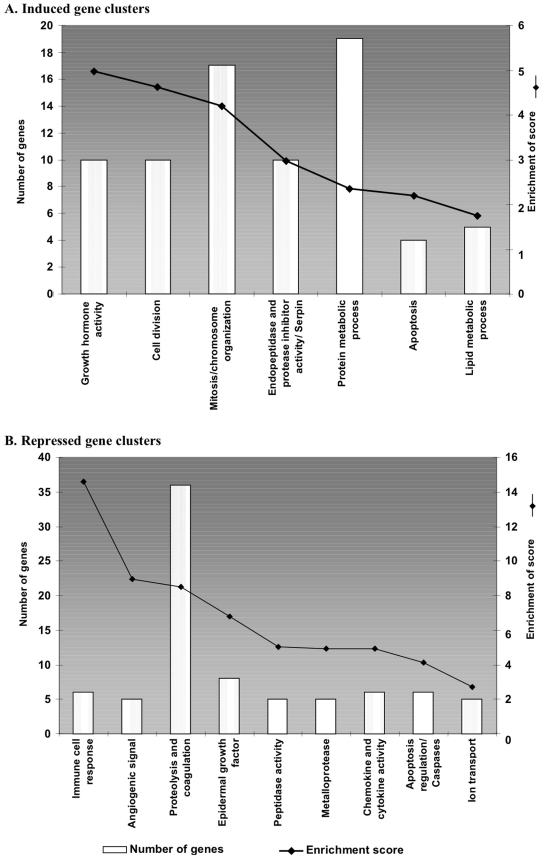
Histograms of gene clusters identified by DAVID for genes induced (A) and repressed (B) in IVF G1/G2. Left ordinate represents the number of genes present in each cluster and the right ordinate represents the enrichment score as defined (see text).

Seven hundred thirty eight genes (corresponding to 1,135 transcripts) induced more than two-fold in the IVF G1/G2 group, were submitted to the DAVID database to attempt a functional clustering [39], [40]. Three hundred ninety one (391) were successfully clustered into 27 functional groups, seven of which were significant according to the threshold defined (Figure 4A, see Materials and Methods). The functional classification of up-regulated genes from the IVF G1/G2 sample is summarized in Figure 4A. A large number of transcripts encoded proteins involved in growth hormone activity, cell division and mitosis, as well as DNA metabolism. These clusters suggest an increased activity of cell growth. The activated DNA metabolism may contribute to explain the massive transcriptional alterations observed on the arrays. An important category relates to protein modifications, essentially by the transcriptional activation of ubiquitin ligases.

A cluster of four apoptotic genes was found including the imprinted gene *Peg3*. This gene is a potential mediator of cell death that cooperates to the p53-mediated cell death pathway. A cluster involved in cellular bioenergetics (lipid metabolism) was also identified.

Figure 4B summarizes the gene clusters that were more than two-fold less abundant after embryo culture in G1/G2 (nine clusters, containing 82 genes, had an enrichment factor above the threshold and were thus analysed further). A large cluster of repressed genes encoded proteins that play a role in immune regulation (orosomucoid, serine peptidase inhibitors and cystatin-f). In addition, two clusters encompassing a total of 41 genes involved in angiogenesis, proteolysis and coagulation were found.

Moreover, modified genes, either up- or down-regulated (1000 transcripts in each category) were grouped into one file for analyzing enriched pathways using DAVID. This selection corresponds to a threshold of transcriptional down-regulation of 2.4 fold or less, and to 2.07 fold or more for induced genes. One thousand seven hundred and eleven (1,711) genes ID were recognized, 163 of which could be incorporated into 13 KEGG pathways. Among the detectable pathways that remain significant after Benjamini-Hochberg correction for multiple testing, we could identify three major groups of genes (Table 2): Complement and coagulation cascades, Natural killer-mediated cell toxicity and Cell adhesion molecules. The main features of these pathways are presented in Supplemental Figures S1, S2 and S3. This finding clearly demonstrates that IVF and embryo culture strongly down-regulate the genes involved in complement pathways, as shown in Supplemental S1. The expression levels of the pivotal molecules C3 and C5 were reduced 3.1 and 2.2 fold, respectively. The complement system behaves as an integrated module of factors, suggesting that one missing element in the cell membrane attack complex may be sufficient to inhibit cell lysis. As such, the sole induction of Complement factor 9, albeit strong (x4.5), is probably not sufficient to promote cell death since the other components are not transcriptionnally increased. Interestingly, in the second pathway significantly enriched (“Natural Killer Cell Mediated Cytotoxicity”, Supplemental S2), there is a very strong overrepresentation of down-regulated genes, suggesting that this pathway is blocked. Finally, the group of genes encoding molecules of cell adhesion is also considerably enriched in down-regulated genes, especially for genes involved in cell-cell signalling in the frame of the immune system (Supplemental S3). In this pathway, many genes are down-regulated more than five-fold (*Icos*, *Itgal*, *Itgb2*, *Ptprc*, *Selplg*). In almost all these cases, the gene expression alterations induced by culture in M16 were slightly stronger (data not shown), but the clusters and pathways identified were the same.

**Table 2:** KEGG pathways identified starting analysis from the 2000 most modified genes in the placenta after IVF and embryo culture.

	Term	Gene Count	%	P-Value	Benjamini
KEGG_PATHWAY	Complement and coagulation cascades	30	1.8	2.3E-12	4.4E-10
KEGG_PATHWAY	Natural killer cell mediated cytotoxicity	29	1.7	0.000015	0.0014
KEGG_PATHWAY	Cell adhesion molecules (CAMs)	32	1.9	0.000026	0.0017
KEGG_PATHWAY	PPAR signaling pathway	16	0.9	0.0042	0.19
KEGG_PATHWAY	C21-Steroid hormone metabolism	6	0.4	0.006	0.21
KEGG_PATHWAY	ECM-receptor interaction	16	0.9	0.016	0.4
KEGG_PATHWAY	Leukocyte transendothelial migration	20	1.2	0.016	0.36
KEGG_PATHWAY	Fructose and mannose metabolism	10	0.6	0.029	0.51
KEGG_PATHWAY	Pentose phosphate pathway	7	0.4	0.031	0.49
KEGG_PATHWAY	Hematopoietic cell lineage	14	0.8	0.056	0.67
KEGG_PATHWAY	Glycolysis/Gluconeogenesis	10	0.6	0.065	0.7
KEGG_PATHWAY	Renin-angiotensin system	5	0.3	0.094	0.8
KEGG_PATHWAY	Bile acid biosynthesis	7	0.4	0.1	0.79

### Genes from the X Chromosome Are Specifically Modified (Essentially Up-Regulated) by IVF Manipulations

**Figure 5:**
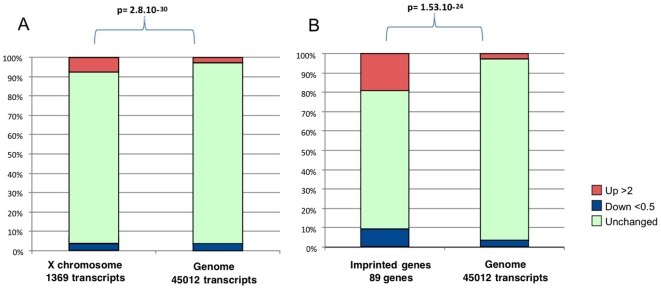
Magnitude of induced (up-regulated more than two-fold), repressed (down-regulated more than two fold) and unchanged genes located on the X-chromosome (A) and imprinted genes (B) compared to the complete set of genes in IVF G1/G2 samples (*P*<0.0001, χ^2^-test).

**Figure 6:**
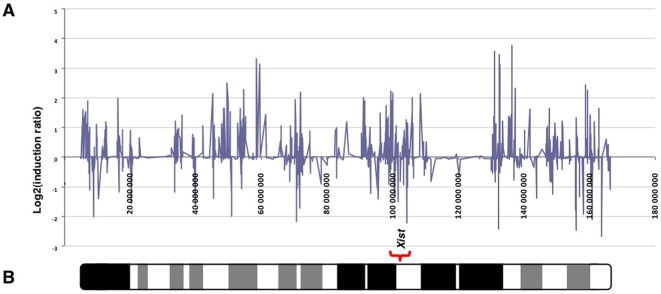
Expression profile of gene expression ratios between in vitro cultured embryos and controls on the X-chromosome do not show a clusterization near the *Xist* gene. **A.** Induction ratios (log2) of genes according to their position on the sequence of X-chromosome in the IVF samples. **B.** Ideogram of *Mus musculus* X-chromosome and localization of *Xist* gene.

We attempted to analyze gene expression modifications according to the chromosome localization. We used the information available from the Affymetrix database to sort the data per chromosome, and we performed a Chi2 test to compare the number of induced/repressed/unmodified genes (at the two-fold threshold) between each chromosome and the rest of the genome (See the Supplemental Table S1). A correction was then applied in order to take into account the multiple testing; with this correction the statistical test was considered significant if below 0.0025. Using this threshold, only the X chromosome was different from the rest of the genome (Figure 5A, χ^2^-test  = 2.8 10^−30^). This skewed proportion was essentially due to the striking abundance of up-regulated genes located on this chromosome (7.4% vs 2.6% for the rest of the genome). By contrast, repressed genes were not different (3.2% vs 3.6% for the rest of the genome). The effect on non pseudoautosomal X genes was apparently not linked to the distance from the X inactivation center (Xic) (Figure 6). This effect was not due to a skewed representation of male and female embryos in the different RNA pools that were used. Since at this early stage, sex determination is starting (Sry is expressed at 10.5 dpc, the possible skews in representation were followed using the expression levels of Y-specific markers such as *UtY*, which were not different between the groups. Moreover, a skewed representation of male and female embryos would have the same consequences on induced and repressed genes, which is not what we observed. Thus, the X-specific transcriptome modifications described here appear genuine.

### Imprinted Genes Are Overrepresented among Modified Genes from the Placenta from IVF and Embryo Culture and Are Mostly Induced Compared to the Rest of the Transcriptome

**Figure 7:**
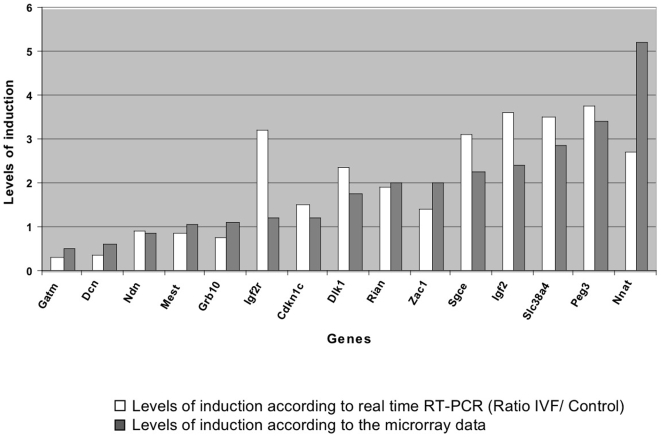
Comparison of levels of induction for 15 genes between real time RT-PCR and microarrays data. Comparison of induction ratios between qRT-PCR and microarray data. The PCR primers were chosen at positions corresponding to Affymetrix features for analysis by qRT-PCR. As clearly shown, there is a very good correlation of the results between the two approaches.

Imprinted genes are known to play important functions in placental development, growth and physiology and are submitted to epigenetic control. Consequently, we analyzed them specifically in our study. Previous expression analysis of a subset of 19 imprinted genes belonging to the imprinted gene network [38] on the placental samples of the present study revealed that manipulated concepti display significantly disturbed levels of gene expression (Fauque et al., submitted). Here, using microarrays, we extend this vision to the complete known set of imprinted genes. Eighty-nine (89) genes known to be imprinted in mice were thus analyzed; seventeen (17) were increased more than two-fold, while eight were reduced more than two-fold by the IVF and embryo culture performed in G1/G2 medium relative to the *in vivo* condition. The microarray results were very similar to those observed by qRT-PCR on a sample of 15 imprinted genes (Figure 7). Compared to the rest of the transcripts, the imprinted genes differed very significantly (χ^2^-test  = 1.53 10^−24^; figure 5B and see the Supplemental Table S2). In addition, it was striking that similarly to the X, the proportion of induced versus repressed genes was inverted compared to the rest of the transcriptome (19.1% induced and 9.0% repressed, versus 2.5% induced and 3.5% repressed in imprinted genes versus the other transcripts, respectively). Furthermore, for the imprinted genes that could be classified according to the expressed allele (paternal or maternal), it appears that while there was the same amount of induced and repressed maternally expressed genes (7 and 6 among 38, respectively), paternally expressed genes appeared primarily induced (7 induced, one repressed among 27 genes).

### Promoter Analysis Reveals a Putative Role of FOXA Transcription Factors in Increasing the Expression of Placental Genes after IVF

**Figure 8:**
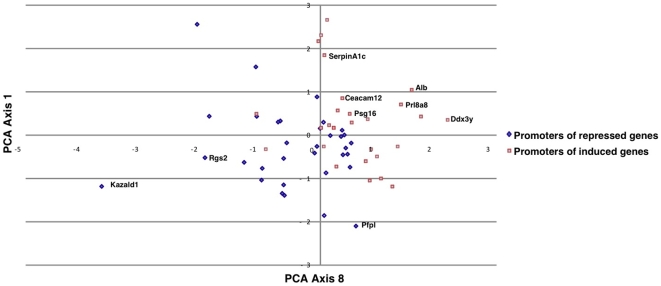
Projections of promoters of induced and repressed genes on axes 1 and 8 after PCPCA (Promoter Content Principal Component Analysis). The axes were defined by PCA from a cross table representing the number of putative transcription factor binding sites in each promoter (60 promoters analyzed corresponding to the 17 most induced and 17 most repressed genes). Among the variable was also added the induction ratio, which permitted to identify the axes that were the most strongly correlated with these ratios (axes 1 and 8). These were chosen for the graphical representation. As clearly shown, there is a very clear dichotomy between induced and repressed promoters when the coordinates calculated for the two axes are used. For clarity, examples of promoters were annotated on the graph.

**Figure 9:**
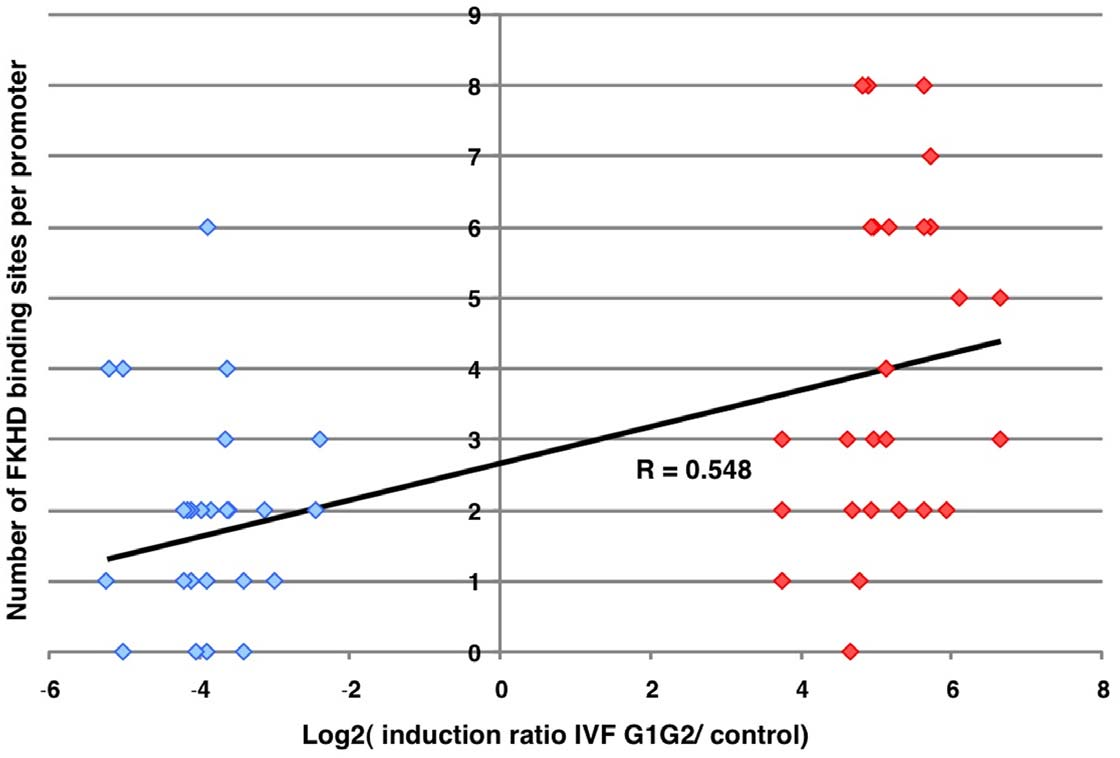
Correlation of representation of the FKHD factors in the promoters of genes highly induced or repressed in the IVF samples. By Student *t*-test analysis, the FKHD binding site was shown to be strikingly overrepresented in induced genes compared to repressed genes. The graph represents promoters of repressed genes on the left side, and of induced genes on the right side. The ordinate corresponds to the number of putative FKHD binding sites found in the promoters using Genomatix™, while the abscissa classifies the promoters according to the Log2 of the induction ratio between the G1/G2 culture conditions versus controls (r = 0.55, *P*<0.0001, *t*-test).

To gain insight into the molecular bases of the aforementioned transcription alterations induced by IVF followed by embryo culture, we analyzed the promoter composition of 34 genes corresponding to the 17 most induced and the 17 most repressed, using Principal Component Analysis (PCA) (for details, see *Materials and Methods* section). Promoter regions were identified using the Gene2Promoter routine of the Genomatix software (http://genomatix.de). The number of putative promoters per gene ranged from one to five. In total, 60 promoters were identified (See the Supplemental Table S3). Then, putative TFBS were detected using Genomatix. In addition to the promoter composition, the level of induction/repression was added to the data matrix file as a variable for calculating the correlation matrix prior to PCA, which made it possible to identify axes 1 and 8 as strongly correlated with this parameter, while the other axes were not (Figure 8). The first axis was also correlated with the CpG composition (data not shown), indicating that genes induced by the IVF/embryo culture protocol have a fairly AT-rich content in their promoters, while repressed genes have a more GC-rich content in their promoters. Again, this is in accordance with the transcriptional induction of Dnmt3a, which can target specific promoters *de novo*, essentially if they are rich in CpG islands [41]. The binding sites of the transcription factors which are correlated with the “Induction Ratio” variable were V$LHXF, V$ATBF, V$HOXF, V$BRNF, V$HOMF on axis 1, and V$RXRF, V$AIRE, V$PLAG, V$FKHD, V$GLIF on axis 8, as referred to in the Genomatix database. The Promoter Content Principal Component Analysis (PCPCA) was thus able to diagnose TFBS that clustered on the promoters of induced genes. It was also interesting to identify whether one (or a few) of these TFBS was differentially represented in the two types of promoters. Student's *t*-test was therefore applied for each TFBS in order to contrast promoter contents between induced and repressed genes, after correction for multiple testing. The most differential binding site was the V$FKHD bound by “forkhead”-containing transcription factors (ForKHeaD), which was strongly over-represented in IVF-induced versus IVF-repressed genes (*P* = 0.001 after correction, Figure 9). Interestingly, this binding site was the only one to remain significant after correction, far beyond the next one V$HNF1 (p = 0.13). We then reanalyze the array data to check for the transcriptional level (induction/repression) of genes belonging to the FOX family, since the TFBS for FOX was overrepresented. *FoxA1*, *FoxA2* and *FoxA3* mRNAs were induced 3.3, 4.4 and 6.2 -fold, respectively. *FoxJ2* gene coding another member of the forkhead transcription factors family was also induced by IVF manipulations (2.2-fold). Following these observations, we moved to expression of human placentas near term (controls and from IVF). Unfortunately, the expression level of these genes is so weak near term that comparisons are impracticable. We also explored a few placentas from the first trimester and could observe a much higher expression of FOXA1, A2 and A3 around 12 weeks of gestation (∼10–15 times, data not shown). These results are not incompatible with the idea that FOXA genes could be involved in human placenta modifications of gene expression when ART is used, but mostly at early stages of gestation when human samples are not accessible for testing.

## Discussion

To the best of our knowledge, our study demonstrates for the first time a substantial alteration of placental gene expression following *in vitro* manipulation techniques of the embryo (above 6% of the transcripts), with a significant excess of repressed genes in the complete transcriptome. There are indications that epigenetic information could be modified in culture and be the cause of long term expressional modifications. Amongst epigenetic mediators, four Dnmt are known, amongst which, Dnmt3a and 3b are known to modify (and not only transmit from one cell generation to the other) epigenetic alterations. Therefore, our findings correlate very well with the overexpression of the *Dnmt3a* gene, encoding the DNA (cytosine-5-)-methyltransferase 3 alpha (x 2.6 in average), which could bring the genome to an overall overmethylation, thus inducing the down-regulation of many genes, especially when their promoter is GC-rich.

The alterations are observed more than 6 days after the *in vitro* manipulations (*in vitro* fertilization and embryo culture) meaning that a ‘memory’ of the injury has been stored during the differentiation of the placenta. These alterations may constitute an important subject of interrogation from the human point of view (for children conceived by ART). This is especially striking, due to the fact that part of the embryo culture of this study was carried out in G1/G2 medium, which is also classically used in humans. However, since the gamete manipulations and fertilization step was performed in M16 medium we can not excluded that this period of stress could contribute very significantly to the large effects seen. Additionally, recent studies have shown that embryo transfer and superovulation could modify imprinted gene expression [42], [43], it would be interesting to perform additional experiments including both variables (superovulation and embryo transfer).

Our data show that *in vitro* fertilization techniques trigger the induction of genes involved in cellular proliferation and cell cycle pathways and an alteration of genes involved in apoptosis pathways. This is probably linked to the invasive properties of trophoblast cells (which promote degradation of the extra-cellular matrix via the production of metalloproteases [44], [45]). Interestingly we observed a decrease in expression of genes involved in the angiogenic signaling, which may explain defects in the future placental function. In addition, repressed gene clusters encompassed genes of the immune system. During pregnancies obtained after IVF and embryo culture, the placenta could therefore be less able to play its protective filtering role against environmental aggressions (bacterial, viral or chemical). This result parallels some of the placental effects of IUGR monitored by transcriptomics in the rat model [46], where the levels of mRNA encoding genes protecting the foeto-placental unit against external aggressions (either xenobiotics or microbial) are reduced.

Interestingly, two categories of genes behave differently from the bulk of the genome: imprinted genes and X-borne genes. Indeed these genes are overall induced, while for the rest of the genome, the reverse situation is observed. Concerning imprinted genes, this observation is consistent with several reports in humans, suggesting that IVF increases the risk of diseases caused by aberrant genomic imprinting, such as Beckwith–Wiedemann syndrome (BWS) [15]–[19], Angelman syndrome (AS) [18], [20], [21] and Silver-Russell syndrome (SRS) [23], [24]. Thus, the epigenetic modifications induced at the early embryo stage [47]–[52] could persist at later stages of development. The observation that early injuries (before implantation) have consequences much later (on the placenta imprinting profile) is consistent with the literature, showing that tissues of trophectoderm origin are unable to restore a correct genomic imprint for the *H19* gene [53] and adequate expression level of this gene [42]. Our results extend the expression anomalies on the complete category of imprinted genes, even showing that they behave differently than the other genes. Interestingly, we observe that the paternally expressed genes are almost systematically induced, which suggests a trend towards favoring placental growth and thus weighing in the balance towards the paternal side of the materno-paternal conflict [54]. Abundant literature suggests that the X inactivation processes share at least partly mechanisms of expression regulation with imprinted genes [55], [56]. Again, our results substantiate this observation. Interestingly, certain X-linked genes are required in mice for normal placental development and for embryonic growth and viability, as the *Cited1* gene [57]. In our experimental groups, this gene was up-regulated (more than 4.5 fold). It is possible that the *Cited1* gene can functionally compensate for the loss of other genes during embryonic development.

We discovered that the promoters of the most strongly activated genes are enriched in forkhead recognition sites, which could be linked to the overexpression of FoxA family genes. These genes could constitute key elements in the observed modifications in gene expression. FOXA proteins are critical for development, differentiation, and metabolism. In particular, mice homozygous for a null mutation in FOXA1 develop a complex phenotype characterized by growth and metabolic abnormalities and display a strong neonatal mortality between days 2 and 14 [58]. In addition, high expression of FOXA1 is commonly observed in prostate tumors. FOXA1 is known to be involved in translating epigenetic signatures since histone H3 lysine 4 dimethylation has been shown to guide FOXA1 recruitment [59], [60]. This may enhance transcription in a controlled fashion with cell type specificity by opening chromatin [61]. Therefore, our results suggest that since IVF results in the transcriptional induction of *FoxA* genes, this constitutes a pathway towards wide changes in gene expression, such as the one observed here. Furthermore, we showed that *FoxJ2* was also up-regulated in IVF samples. *FoxJ2* over-expression using transgenic mice has a negative effect on embryonic development [62].

Our data on the transcriptomic consequences of ART manipulations in the mouse model at the post-implantation stage establish expressional disturbances in placental tissues that consequently modify trophic adjustments and might be connected to birth weight, obesity, type II diabetes or hypertension later in life. Such differences have the potential to influence long-term patterns of gene expression that might be associated with increased risk of many human diseases. Additionally, our results suggest that a family of transcriptional regulators (FOXA) may be involved in the response to the injury in the placenta. In future studies, analysis of permanent tissues (such as liver, kidney, brain) in mice following ART will have to be performed.

## Materials and Methods

### Animals

Five- to six-week-old F1 (C57BL/6/CBA) females and eight- to nine-week-old F1 males (Charles River Laboratories, L'Arbresle, France) were maintained in an animal facility at normal temperature (21–23°C) and 14 h light/10 h dark photoperiods with free access to water and food. Procedures for handling and experimentation followed ethical guidelines established by the Federation of European Laboratory Animal Science Associations.

### Experimental Design

All female mice were superovulated and were divided into three different experimental groups (IVF in M16 medium, IVF in G1/G2 medium groups versus control group). In the control group, blastocysts were collected after *in vivo* fertilization and *in vivo* development. In the two IVF groups, blastocysts were obtained after *in vitro* fertilization and development. The embryos from IVF were cultured either in M16 medium (IVF M16 group; Sigma-Aldrich, Lyon, France) or in sequential G1/G2 medium (G1.G2 group; JCD Laboratories, Lyon, France) respectively. All embryos were then transferred to pseudopregnant females.

#### Superovulation

Females were superovulated by intraperitoneal (i.p.) injection of 8 IU (0.1 ml) Pregnant Mare Serum Gonadotropin (PMSG, Chronogest; Intervet, Beaucouzé, France), followed 47 h later by an i.p. injection of 5 IU (0.1 ml) of human Chorionic Gonadotropin (hCG, Chorulon; Intervet).

#### *In vitro* fertilization and embryo culture

Thirteen hours post-hCG, cumulus-oocyte complexes were recovered from oviducts in M2 medium (Sigma-Aldrich) supplemented with 7 mg/ml of BSA (Sigma-Aldrich). After rinsing in M16 medium, cumulus-oocyte complexes were kept in the incubator (37°C, 5.5% CO2 in air) in 100 µl drops of M16 medium covered with paraffin oil (Vitrolife). Spermatozoa were collected from the cauda epididymis and capacitated for 90 min in M16 medium supplemented with 7 mg/ml of BSA at 37°C and 5.5% CO_2_. Oocytes were inseminated 15 h post-hCG with 10^6^ spermatozoa. Fertilized eggs (23 h post-hCG) as determined by the presence of two pronuclei were then transferred to 30 µl drops of fresh medium covered with paraffin oil. To analyze the embryo culture medium effects, two different media were tested: the M16 medium and the sequential G1/G2 medium [63] containing amino acids. The embryo culture was conducted up to the blastocyst stage at 37°C and 5.5% CO_2_. The culture medium was changed daily.

#### Collection of blastocysts

Recipient F1 females were mated individually with F1 males after hCG injection. The following morning, females were checked for vaginal copulation plug. Embryos at the blastocyst stage were obtained by flushing the uterus 3.5 days p.c. with M2 medium supplemented with BSA (7 mg/ml).

#### Embryo transfer recipients

F1 (C57BL/6/CBA) females of at least 6 weeks of age were mated to vasectomized F_1_ males 1 day prior to embryo transfer. The morning after mating, females were checked for the presence of a vaginal plug, and this was considered as day 0.5 of pseudopregnancy. A total of 40 and 51 embryos (at early, non expanded and fully expanded blastocyst stages, classification described in our previous study, [52]) were transferred to the uteri of pseudopregnant females of control and IVF groups respectively, on pseudopregnant d3.5 according to standard procedures [42]. On day 10.5, females were sacrificed and concepti with a normal development (stage 17–18 as defined by Theiler et *al*., [64]) were immediately collected (Supplemental Table S4). After removal of yolk sac and embryos, placentas were dropped in 250 µl of Trizol reagent (Invitrogen Life Technology, Cergy, France), snap-frozen and stored at –80°C until further use.

### RNA Preparation, cDNA Synthesis, and Microarray Hybridizations

#### RNA preparation

Total RNA was extracted from a pool of five placental samples coming from at least two litters from the same group with Trizol (Invitrogen Life Technology) according to the manufacturer's instructions and treated with DNase I to eliminate genomic DNA contamination. The integrity of the total RNA was investigated using the Bioanalyser 2100 and the RNA 6000 nano LabChip kit (Agilent Technologies). Only total RNA samples with a RIN number >8 were used.

#### cRNA synthesis and probe array hybridization

cRNA synthesis was performed using 3 µg of total RNA using the GeneChip Expression 32 Amplification One-Cycle Target Labelling, purified and fragmented before hybridization onto the mouse Affymetrix 430.2.0 GeneChips (Affymetrix Inc., Santa Clara, CA, USA) according to the manufacturer's protocol. Briefly, total RNA was first reverse transcribed using a T7-Oligo(dT) promoter primer in the first-strand cDNA synthesis reaction. Following RNase H-mediated second-strand cDNA synthesis, the double-stranded cDNA was purified and served as a template in the subsequent *in vitro* transcription (IVT) reaction. The IVT reaction was carried overnight at 37°C using the T7 RNA polymerase and a biotinylated nucleotide analog/ribonucleotide mix to generate labeled complementary RNA (cRNA). The biotinylated cRNA targets were then cleaned up, fragmented, and controlled on bioanalyzer 2100 before hybridization on murine GeneChip 430 2.0 expression arrays. After washing and staining using the specific protocols on the fluidics station FS450 (Affymetrix, Inc.), the chips were scanned with the GeneChip Scanner 3000 according to the manufacturer's instructions (GeneChip Analysis Technical Manual, www.affymetrix.com).

### Microarray Gene Expression Data Analysis

The quality controls of the hybridization were performed using the MAS5 algorithm (implemented in GCOS software from Affymetrix) to determine the percentage of present probe sets for each chip of the study: close values were obtained in the range of expected values for high quality data according to Affymetrix standards (52.2%, 53.6%, 53.1% for control, M16 and G1/G2, respectively).

The dataset was then analyzed with GeneSpring (software version 7.2 -Agilent Technologies, France) for data analysis. The robust multiarray analysis algorithm (RMA, GeneSpring; Agilent Technologies) was applied to the data for background adjustment, normalization, and log2 transformation of perfect match values [65].

#### Functional clustering by DAVID (Database for Annotation and Visualization and Integrated Discovery)

Two lists of genes induced or repressed twice or more in the placentas were submitted to the DAVID database [40]. Briefly, DAVID clusterizes genes from a list according to a series of keywords common to several genes from the list. The proportion of each keyword from the gene list submitted is compared with the proportion in the whole genome, making it possible to compute a *P* value. Enrichment values are then calculated as the geometric mean of the inverse log of each *P* value. These enrichment scores depend on the number of genes present in the list. Therefore, we used a simulation to define minimal enrichment thresholds enabling us to identify gene clusters given by DAVID for further analysis, as previously described [46]. The groups were considered significant if they showed an enrichment value greater than 1.75 and 2.70 for induced and repressed gene clusters, respectively.

#### Promoter content principal component analysis (PCPCA)

A set of genes presenting an average of at least two-fold induction/repression calculated from the different probes specific for each gene spotted on the Affymetrix chip was selected for promoter analysis. Among these genes, the 17 most induced genes and the 17 most repressed were chosen. Putative promoter regions were automatically identified by using the Gene2Promoter routine of Genomatix (Munich, Germany) software (http://genomatix.de). The putative transcription factor binding sites (TFBS) were detected by using the Gene2Promoter function of the Genomatix software (Genomatix.de). The putative TFBS composition of 27 promoters from induced genes and 33 promoters from repressed genes was used to generate a correlation matrix of 153 rows [all the 152 putative TFBS detected by Genomatix and the log_2_ (induction ratio) for each gene] and 60 columns (See the Supplemental Table S3). This table was used to generate a correlation matrix, which allowed us to identify Eigen values and principal components, using the principal component utility from the StatistiXL software (http://www.statistixl.com). The Eigen values were classified in decreasing order to define the corresponding axes, the first axis collecting the highest percentage of information.

#### KEGG pathways

Differentially expressed genes in IVF versus control were classified in pathways established by the Kyoto Encyclopedia of Genes and Genomes (KEGG) pathways [66].

### Quantitative RT–PCR Analysis in Mouse

The analysis of imprinted gene expression was performed in a previous study (Fauque et al., submitted) according to published protocols [38].

### Human Patients

Placentas from control and ART patients were collected from the Saint Vincent de Paul maternity hospital, Paris. Informed consent was obtained from the patients under the auspices of a CPP (Committee for Patient Protection, Paris Cochin), in conformity with the Helsinki declaration. Total placental RNA was obtained from isolated placental villi dissected free from maternal membranes in HBSS on the maternal side as previously described [67]. Fragments were frozen in Trizol™ prior to RNA extraction, which was carried out according to previously published protocols [67].

#### RNA Extraction and cDNA synthesis in Human

Total RNA was extracted using Trizol Reagent (Invitrogen) in accordance with the manufacturer's instructions. cDNA synthesis was performed using Superscript cDNA synthesis kit (Invitrogen).

#### Quantitative RT-PCR analysis in Human

Quantitative RT PCR was performed using a Light Cycler (Roche) and the Invitrogen kit (SYBR®). The primers for cDNA amplification (see Supplemental Table S5) were chosen using the PRIMER3 software (http://frodo.wi.mit.edu/cgi-bin/primer3) with SDHA as one of the best reference genes for placental structures [68].

### Statistics

Student's *t* -test, ANOVA and Mann–Whitney nonparametric tests were used throughout the study, with a *P<*0.05 threshold considered significant.

## Supporting Information

Figure S1 Coagulation and complement pathway regulated by IVF (mammalian KEGG pathway). Induced genes are represented in red, repressed genes in blue. This representation makes it possible to identify relevant ways by which IVF associated to embryo culture in G1/G2 medium may affect the coagulation.
(2.08 MB DOC)

Figure S2 Natural cell killer pathways are down-regulated in the placental following IVF (mammalian KEGG pathway). Induced genes are represented in red, repressed genes in blue. This representation makes it possible to identify relevant ways by which IVF associated to embryo culture in G1/G2 medium may affect the coagulation.
(0.13 MB DOC)

Figure S3 Cell-cell interactions pathways are modified in the placental following IVF (mammalian KEGG pathway). Induced genes are represented in red, repressed genes in blue. This representation makes it possible to identify relevant ways by which IVF associated to embryo culture in G1/G2 medium may affect the coagulation.
(0.14 MB DOC)

Table S1 Comparison of the number of induced/repressed/unmodified genes (at the two fold threshold) between each chromosome and the rest of the genome with the statistical Chi2 test.
(0.06 MB DOC)

Table S2 Induction ratios of imprinted genes (red and blue boxes for up-regulated and down-regulated genes respectively) in the placenta after IVF (IVF M16 and IVF G1/G2)
(0.10 MB DOC)

Table S3 Composition of the promoters from the most induced (Red) and the most repressed genes (Blue). The first row corresponds to the accession numbers of the various promoters analyzed obtained from Genomatix, the second to the induction ratio, the third to the base2 logarithm of this ratio. Follow locus names and gene names in the next two rows. The different DNA binding sites are listed in the left column and classified from the most significant to the least (Statistical tests - Student with and without Bonferronni corrections and correlation values in black when significant) are at the right of the table. The values inside the table correspond to the number of putative Transcription Factor binding sites found by Genomatix in each promoter.
(0.34 MB DOC)

Table S4 Litter characteristics of control and IVF litters following embryo transfer.
(0.04 MB DOC)

Table S5 Primers for Quantitative PCR in Human.
(0.03 MB DOC)
