# Supplementary material for: In Vitro Fertilization and Embryo Culture Strongly Impact the Placental Transcriptome in the Mouse Model
Source: PLoS One. 2010 Feb 15;5(2):e9218. doi: 10.1371/journal.pone.0009218 (PMC2821408; doi:10.1371/journal.pone.0009218)
Supplement: Figure S1 — Coagulation and complement pathway regulated by IVF (mammalian KEGG pathway). Induced genes are represented in red, repressed genes in blue. This representation makes it possible to identify relevant ways by which IVF associated to embryo culture in G1/G2 medium may affect the coagulation. (2.08 MB DOC) [file pone.0009218.s001.doc]

**Supplemental Figure S1 – Coagulation and complement pathway regulated by IVF** (mammalian KEGG pathway). Induced genes are represented in red, repressed genes in blue. This representation makes it possible to identify relevant ways by which IVF associated to embryo culture in G1/G2 medium may affect the coagulation.

**Number of genes**

**Enrichment score**

Unchanged

Down <0.5

Up >2
