# Supplementary material for: In Vitro Fertilization and Embryo Culture Strongly Impact the Placental Transcriptome in the Mouse Model
Source: PLoS One. 2010 Feb 15;5(2):e9218. doi: 10.1371/journal.pone.0009218 (PMC2821408; doi:10.1371/journal.pone.0009218)
Supplement: Figure S3 — Cell-cell interactions pathways are modified in the placental following IVF (mammalian KEGG pathway). Induced genes are represented in red, repressed genes in blue. This representation makes it possible to identify relevant ways by which IVF associated to embryo culture in G1/G2 medium may affect the coagulation. (0.14 MB DOC) [file pone.0009218.s003.doc]

**Supplemental Figure S3 – Cell-cell interactions pathways are modified in the placental following IVF** (mammalian KEGG pathway). Induced genes are represented in red, repressed genes in blue. This representation makes it possible to identify relevant ways by which IVF associated to embryo culture in G1/G2 medium may affect the coagulation.

Unchanged

Down <0.5

Up >2
