# Supplementary material for: In Vitro Fertilization and Embryo Culture Strongly Impact the Placental Transcriptome in the Mouse Model
Source: PLoS One. 2010 Feb 15;5(2):e9218. doi: 10.1371/journal.pone.0009218 (PMC2821408; doi:10.1371/journal.pone.0009218)
Supplement: Table S1 — Comparison of the number of induced/repressed/unmodified genes (at the two fold threshold) between each chromosome and the rest of the genome with the statistical Chi2 test. (0.06 MB DOC) [file pone.0009218.s004.doc]

| **Supplemental Table S1.** Comparison of the number of induced/repressed/unmodified genes (at the two fold threshold) between each chromosome and the rest of the genome with the statistical Chi2 test | | | | | | | | | | | | | | | | | | | | | | | |
| --- | --- | --- | --- | --- | --- | --- | --- | --- | --- | --- | --- | --- | --- | --- | --- | --- | --- | --- | --- | --- | --- | --- | --- |
|  | **MMU1** | **MMU2** | **MMU3** | **MMU4** | **MMU5** | **MMU6** | **MMU7** | **MMU8** | **MMU9** | **MMU10** | **MMU11** | **MMU12** | **MMU13** | **MMU14** | **MMU15** | **MMU16** | **MMU17** | **MMU18** | **MMU19** | **MMUX** | **All ORFs** |  | **Genome** |
| **Induced (No.)** | 81 | 69 | 59 | 59 | 65 | 62 | 82 | 38 | 66 | 44 | 54 | 49 | 62 | 35 | 31 | 57 | 28 | 33 | 34 | 101 | 1109 | 0,02565942 | 1135 |
| **Repressed (No.)** | 108 | 105 | 97 | 95 | 98 | 98 | 108 | 66 | 82 | 53 | 119 | 81 | 57 | 50 | 65 | 51 | 73 | 33 | 76 | 44 | 1559 | 0,03607126 | 1586 |
| **Unchanged (No.)** | 2515 | 3307 | 2073 | 2435 | 2547 | 2275 | 2684 | 1997 | 2158 | 1952 | 3205 | 1568 | 1670 | 1519 | 1643 | 1410 | 1776 | 1199 | 1395 | 1224 | 40552 | 0,93826932 | 42380 |
|  | 2704 | 3481 | 2229 | 2589 | 2710 | 2435 | 2874 | 2101 | 2306 | 2049 | 3378 | 1698 | 1789 | 1604 | 1739 | 1518 | 1877 | 1265 | 1505 | 1369 | 43220 |  | 45101 |
| **Expected (No.)** | 68.0481586 | 87.6019379 | 56.0944325 | 65.1540986 | 68.199153 | 61.2785748 | 72.3263342 | 52.8732179 | 58.0321944 | 51.5645995 | 85.0098667 | 42.731425 | 45.0215073 | 40.3658455 | 43.7632203 | 38.201592 | 47.2360923 | 31.83466 | 37.8744374 | 34.4518969 | 1109 |  |  |
|  | 95.087559 | 122.411166 | 78.3839383 | 91.0435245 | 95.2985521 | 85.6280349 | 101.065697 | 73.882752 | 81.0916831 | 72.0541451 | 118.789118 | 59.7110485 | 62.9111106 | 56.4054899 | 61.1528347 | 53.3812554 | 66.0056761 | 44.4843795 | 52.9241037 | 48.1415933 | 1559 |  |  |
|  | 2540.86428 | 3270.9869 | 2094.52163 | 2432.80238 | 2546.50229 | 2288.09339 | 2700.60797 | 1974.24403 | 2166.87612 | 1925.38126 | 3174.20102 | 1595.55753 | 1681.06738 | 1507.22866 | 1634.08394 | 1426.41715 | 1763.75823 | 1188.68096 | 1414.20146 | 1286.40651 | 40552 |  |  |
| Chi2/anonymous ORF | 0.10635752 | 0.0329912 | 0.09103938 | 0.68550328 | 0.89281811 | 0.39239314 | 0.3922396 | 0.07110632 | 0.565381 | 0.03845634 | 0.00301098 | 0.01118843 | 0.02973009 | 0.46472158 | 0.13446011 | 0.00845676 | 0.01316956 | 0.21255365 | 0.00470426 | 2.2482E-29 | 1 |  |  |
| Bonferronni correction | 2.12715031 | 0.65982394 | 1.82078752 | 13.7100657 | 17.8563623 | 7.8478629 | 7.84479207 | 1.42212631 | 11.3076199 | 0.76912681 | 0.06021954 | 0.22376863 | 0.59460179 | 9.29443158 | 2.68920216 | 0.16913517 | 0.26339124 | 4.25107296 | 0.09408519 | 4.4964E-28 |  |  |  |
|  |  |  |  |  |  |  |  |  |  |  |  |  |  |  |  |  |  |  |  |  |  |  |  |
| Contingence Test | |  |  |  |  |  |  |  |  |  | 0.00188182 |  |  |  |  |  |  |  |  | 2.8512E-30 |  |  |  |
| Bonferronni correction | |  |  |  |  |  |  |  |  |  | 0.03763632 |  |  |  |  |  |  |  |  | 5.7023E-29 |  |  |  |
