# Supplementary material for: In Vitro Fertilization and Embryo Culture Strongly Impact the Placental Transcriptome in the Mouse Model
Source: PLoS One. 2010 Feb 15;5(2):e9218. doi: 10.1371/journal.pone.0009218 (PMC2821408; doi:10.1371/journal.pone.0009218)
Supplement: Table S2 — Induction ratios of imprinted genes (red and blue boxes for up-regulated and down-regulated genes respectively) in the placenta after IVF (IVF M16 and IVF G1/G2) (0.10 MB DOC) [file pone.0009218.s005.doc]

| **Supplemental Table S2 : Induction ratios of imprinted genes (red and blue boxes for up-regulated and down-regulated genes respectively) in the placenta after IVF (IVF M16 and IVF G1/G2)** | | |
| --- | --- | --- |
| **Imprinted Genes** | **IVF M16** | **IVF G1/G2** |
| **4930524O08Rik *(A19)*** | 1.00 | 1.00 |
| ***Air (Igf2rAS)*** | 0.94 | 1.00 |
| ***Air (Igf2rAS)*** | 2.07 | 1.53 |
| ***Ampd3*** | 0.29 | 0.32 |
| ***Apoc2*** | 6.90 | 11.71 |
| ***Apoe*** | 1.86 | 3.14 |
| ***Asb4*** | 1.26 | 2.46 |
| ***Ascl2 (Mash2)*** | 1.73 | 1.36 |
| ***Ascl2 (Mash2)*** | 1.84 | 1.52 |
| ***Ascl2 (Mash2)*** | 1.57 | 1.55 |
| ***Atp10a (Atp10c, Pfatp)*** | 0.26 | 0.31 |
| ***Axin1, Fused, Fu*** | 1.35 | 1.35 |
| ***Calcr*** | 0.93 | 0.96 |
| ***Cd81 (Tapa1)*** | 0.34 | 0.48 |
| ***Cdkn1c (p57, Kip2)*** | 1.19 | 1.23 |
| ***Commd1 (Murr1)*** | 0.94 | 1.00 |
| ***Copg2*** | 0.94 | 1.04 |
| ***Copg2as2 (Mit1, Lb9)*** | 0.13 | 0.18 |
| ***Dcn*** | 0.54 | 0.66 |
| ***Ddc*** | 4.17 | 7.78 |
| ***Dhcr7*** | 0.26 | 0.33 |
| ***Dlk1 (pref-1, Ly107, FA1, SCP1, Zog, Peg9)*** | 1.63 | 1.77 |
| ***Esx1, Spx1*** | 1.22 | 1.22 |
| ***Gabra5*** | 0.92 | 0.96 |
| ***Gabrb3*** | 0.93 | 0.96 |
| ***Gabrg3*** | 0.96 | 0.98 |
| ***Gatm*** | 0.56 | 0.54 |
| ***Gnas (Gs-alpha), Gnasxl, Nesp, Nespas, F7*** | 0.78 | 0.92 |
| ***Grb10 (Meg1)*** | 1.58 | 1.15 |
| ***H19, Mirn675*** | 1.19 | 1.24 |
| ***Igf2*** | 2.16 | 2.41 |
| ***Igf2as*** | 1.89 | 1.87 |
| ***Igf2r*** | 1.55 | 1.11 |
| ***Impact*** | 1.13 | 0.73 |
| ***Inpp5f_v2*** | 1.90 | 1.98 |
| ***Ins1, insulin I (conflicting data)*** | 1.00 | 1.00 |
| ***Kcnq1 ( Kvlqt1, Kcna9)*** | 2.53 | 1.37 |
| ***Kcnq1ot1 (Kvlqt-as)*** | 1.00 | 1.00 |
| ***Klf14*** | 1.39 | 1.39 |
| ***Magel2*** | 1.53 | 1.39 |
| ***Mas1 (disputed)*** | 1.09 | 0.82 |
| ***Meg3 (Gtl2)*** | 3.67 | 2.28 |
| ***Mest (Peg1)*** | 1.02 | 1.11 |
| ***Mirg (microRNA cluster: miR-411, miR-380, miR-376b, miR-376,miR-134, miR-154, miR-410)*** | 2.04 | 2.17 |
| ***Mkrn3 (Zfp127, Znf127)*** | 1.01 | 1.00 |
| ***Nap1l4 (Nap2)*** | 1.25 | 1.25 |
| ***Nap1l5*** | 1.00 | 1.00 |
| ***Ndn (necdin)*** | 0.68 | 0.97 |
| ***Nf1*** | 1.00 | 1.00 |
| ***Nnat (Peg5, Neuronatin)*** | 2.19 | 5.24 |
| ***Osbpl5 (Obph1)*** | 0.24 | 0.38 |
| ***Pax1, Un(s)*** | 1.00 | 1.00 |
| ***Peg10*** | 1.90 | 1.79 |
| ***Peg12 (Frat3)*** | 0.87 | 1.00 |
| ***Peg13*** | 0.92 | 0.97 |
| ***Peg3, Pw1*** | 5.01 | 3.46 |
| ***Phlda2 (Tssc3, Ipl)*** | 1.47 | 1.78 |
| ***Plagl1, Zac1, Lot1*** | 1.88 | 2.05 |
| ***Pon1 (Paraoxonase 1)*** | 1.00 | 1.00 |
| ***Pon2 (paraoxonase 2)*** | 0.99 | 1.00 |
| ***Pon3 (paraoxonase 3)*** | 0.42 | 0.56 |
| ***Ppp1cc, protein phosphatase 1, catalytic subunit, gamma isoform, Pp1c gamma*** | 0.78 | 0.83 |
| ***Ppp1r9a (neurabin)*** | 3.04 | 2.92 |
| ***Rasgrf1 (Grf1, Cdc25Mm, p190)*** | 1.17 | 1.00 |
| ***Rb1*** | 3.98 | 1.91 |
| ***Rhox5 (Pem)*** | 1.75 | 1.99 |
| ***Rian (C/D snoRNA cluster: MBII-343, MBII-426, MBII-78, MBII-19, MBII-48, MBII-49, miR-370)*** | 3.39 | 2.72 |
| ***Sfmbt2*** | 1.62 | 1.29 |
| ***Sgce*** | 4.28 | 2.30 |
| ***Slc22a18 (Slc22a1l, Orctl2, Impt1, Itm, Tssc5, Bwscr1a)*** | 0.97 | 1.50 |
| ***Slc22a2, Orct2*** | 1.76 | 2.21 |
| ***Slc22a3, Orct3, EMT*** | 1.72 | 1.72 |
| ***Slc38a4 (1110012E16Rik, 1700012A18Rik, Ata3)*** | 4.50 | 2.87 |
| ***Snrpn, Snurf, IPW, MBII-436, MBII-13, Pwcr1 (MBII-85), MBII-52, Ube3a-AS*** | 1.63 | 1.33 |
| ***Tfpi2*** | 1.00 | 1.25 |
| ***Th*** | 1.00 | 1.00 |
| ***Tnfrsf23 (Tnfrh1)*** | 0.41 | 0.44 |
| ***Tsix*** | 0.96 | 0.99 |
| ***Tspan32 (Phemx, Tssc6)*** | 0.94 | 0.94 |
| ***Tssc4*** | 0.93 | 1.00 |
| ***Ube3a*** | 1.11 | 1.31 |
| ***Usp29 (Ocat)*** | 1.05 | 1.05 |
| ***Xist*** | 0.28 | 0.40 |
| ***Xlr4b*** | 2.40 | 2.20 |
| ***Xlr4c*** | 1.00 | 1.00 |
| ***Zim1*** | 2.55 | 3.06 |
| ***Zim2*** | 0.96 | 0.98 |
| ***Zim3*** | 1.00 | 1.00 |
| ***Zrsr1 (U2af1-rs1, U2afbp-rs, D11Ncvs75)*** | 1.08 | 1.17 |
