# Supplementary material for: In Vitro Fertilization and Embryo Culture Strongly Impact the Placental Transcriptome in the Mouse Model
Source: PLoS One. 2010 Feb 15;5(2):e9218. doi: 10.1371/journal.pone.0009218 (PMC2821408; doi:10.1371/journal.pone.0009218)

Supplemental Table S3 : Composition of the promoters from the most induced (Red) and the most repressed genes (Blue). The first row corresponds to the accession numbers of the various promoters analyzed obtained from Genomatix, the second to the induction ratio, the third to the base2 logarithm of this ratio. Follow locus names and gene names in the next two rows. The different DNA binding sites are listed in the left column and classified from the most significant to the least (Statistical tests – Student with and without Bonferronni corrections and correlation values in black when significant) are at the right of the table. The values inside the table correspond to the number of putative Transcription Factor binding sites found by Genomatix in each promoter.


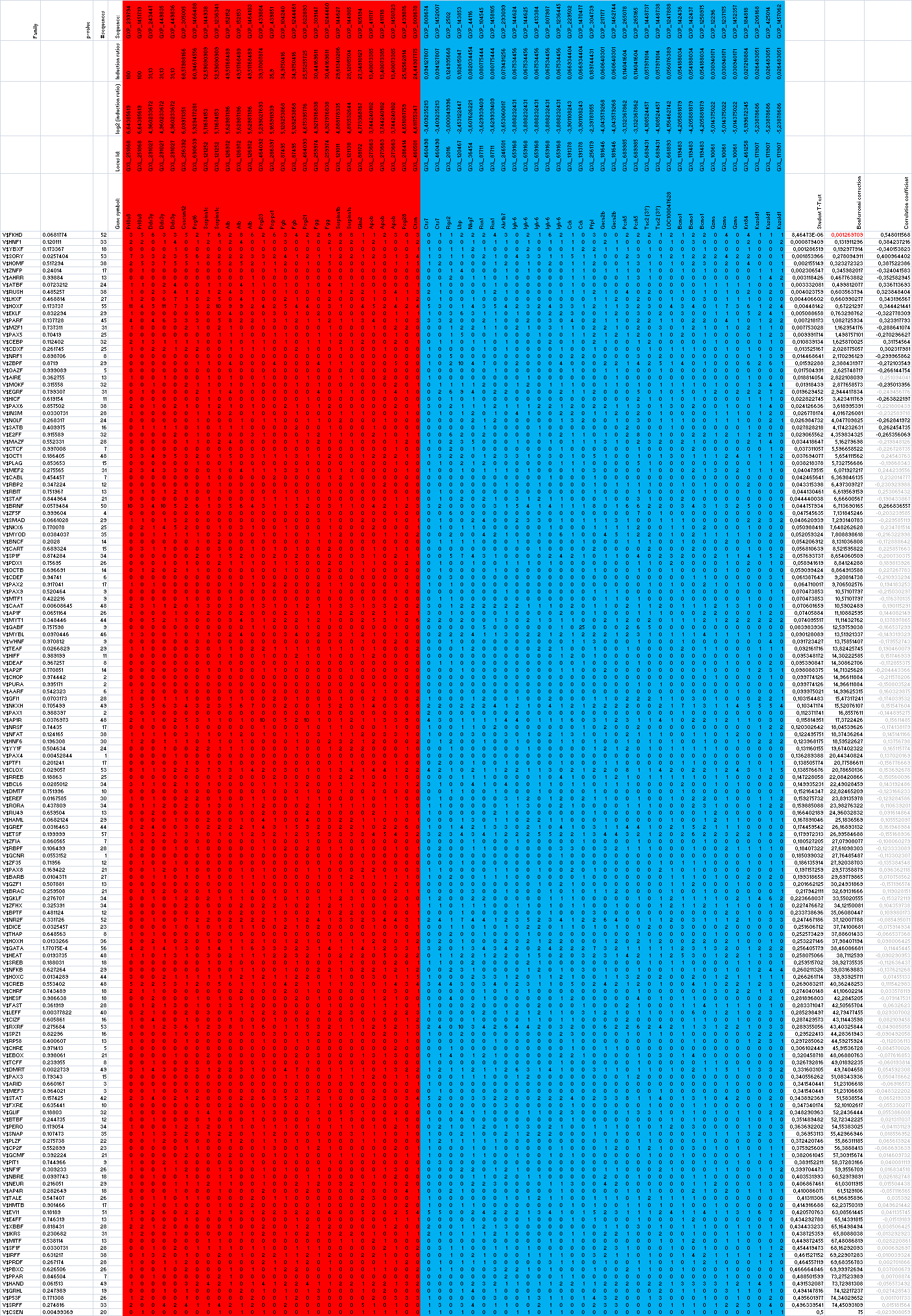


Table S7’ : Coordinates of the different transcription factor binding sites (TFBS) on the 5 axes for which there is a significant correlation by two different statistical tests with the induction ratio (complete dataset available on request). This type of analysis is able to identify several putative TFBS associated with the induction ratio, while the one above (S1) makes a separate statistical analysis for every binding site. For axes 1, 8, 30, 31 and 41, the induction ratio is ranked 150^th^ , 148^th^ , 149^th^ ,1^st^ and 150^th^ , respectively amongst 153 variables, which shows the link between this parameter and each of these axes. Interestingly, the FKHD binding site ranks 141th, 152th, 129^th^ , 10^th^ and 69^th^, respectively amongst the 153 variables. Therefore, on the four first axes, there is a very good correlation between the induction ratios and the presence of FKHD binding sites, whereas, for the fifth axis (#41), there is no correlation. By contrast, this axis is determined by the V$CP2F, V$CDXF, V$TEAF which are correlated with the induction ratio, while V$PBXC and V$STAF are negatively associated with the induction ratio.


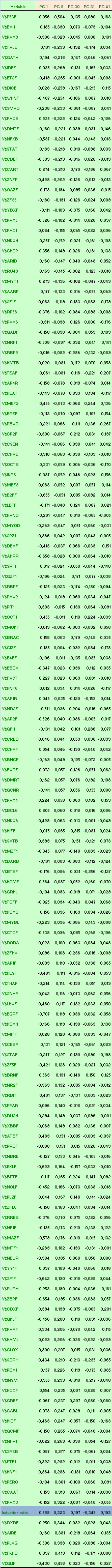

Supplement: Table S3 — Composition of the promoters from the most induced (Red) and the most repressed genes (Blue). The first row corresponds to the accession numbers of the various promoters analyzed obtained from Genomatix, the second to the induction ratio, the third to the base2 logarithm of this ratio. Follow locus names and gene names in the next two rows. The different DNA binding sites are listed in the left column and classified from the most significant to the least (Statistical tests - Student with and without Bonferronni corrections and correlation values in black when significant) are at the right of the table. The values inside the table correspond to the number of putative Transcription Factor binding sites found by Genomatix in each promoter. (0.34 MB DOC) [file pone.0009218.s006.doc]
