# Supplementary material for: In Vitro Fertilization and Embryo Culture Strongly Impact the Placental Transcriptome in the Mouse Model
Source: PLoS One. 2010 Feb 15;5(2):e9218. doi: 10.1371/journal.pone.0009218 (PMC2821408; doi:10.1371/journal.pone.0009218)
Supplement: Table S4 — Litter characteristics of control and IVF litters following embryo transfer. (0.04 MB DOC) [file pone.0009218.s007.doc]

**Supplemental Table S4. Litter characteristics of control and IVF litters following embryo transfer.**

|  | No. of blastocysts transferred | No. of implantation sites^a^ (%) | No. of resorption sites (%) | Numbers collected embryos^b^ (%) |
| --- | --- | --- | --- | --- |
| **Control litters** |  |  |  |  |
| Litter 1 | 11 | 7 | 3 | 4 |
| Litter 2 | 11 | 7 | 1 | 6 |
| Litter 3 | 9 | 6 | 0 | 6 |
| Litter 4 | 9 | 7 | 1 | 6 |
| *Total* | *40* | *27 (67.5)* | *5 (18.5)* | *22 (55.0)* |
|  |  |  |  |  |
| **IVF litters** |  |  |  |  |
| Litter 1 (M16) | 10 | 7 | 4 | 3 |
| Litter 2 (M16) | 11 | 9 | 6 | 3 |
| Litter 3 (M16) | 10 | 8 | 5 | 3 |
| Litter 4 (G1/G2) | 9 | 7 | 6 | 1 |
| Litter 5 (G1/G2) | 11 | 9 | 5 | 4 |
| Litter 6 (G1/G2) | 10 | 8 | 5 | 3 |
| *Total* | *61* | *48 (78.7)* | *31 (64.6)** | *17 (27.9)* |

^a^ Implantation sites included viable embryos and resorption sites. Implantation rate was defined as the ratio between number of implantation sites and the number of blastocysts transferred.

^b^ Collected embryos were embryos having a correct developmental stage without abnormal or retarded embryo according to the Theiler classification.

**P* < 0.001, Chi2-test, control versus IVF groups.
