# Supplementary material for: In Vitro Fertilization and Embryo Culture Strongly Impact the Placental Transcriptome in the Mouse Model
Source: PLoS One. 2010 Feb 15;5(2):e9218. doi: 10.1371/journal.pone.0009218 (PMC2821408; doi:10.1371/journal.pone.0009218)
Supplement: Table S5 — Primers for Quantitative PCR in Human. (0.03 MB DOC) [file pone.0009218.s008.doc]

| **Supplemental Table S5. Primers for Quantitative PCR in Human.** | | |
| --- | --- | --- |
| Gene name | Forward primer | Reverse primer |
| FOXA1 | GAAGATGGAAGGGCATGAAA | GCCTGAGTTCATGTTGCTGA |
|  |  |  |
| FOXA2 | TTTAAACTGCCATGCACTCG | GTTGCTCACGGAGGAGTAGC |
|  |  |  |
| FOXA3 | CTGGCCGAGTGGAGCTACTA | AGGGGGATAGGGAGAGCTTA |
|  |  |  |
| SDHA | TACAAGGTGCGGATTGATGA | GCAACAGAAGAAGCCCA |
